# Adoption, implementation and sustainability of school-based physical activity and sedentary behaviour interventions in real-world settings: a systematic review

**DOI:** 10.1186/s12966-019-0876-4

**Published:** 2019-12-02

**Authors:** Samuel Cassar, Jo Salmon, Anna Timperio, Patti-Jean Naylor, Femke van Nassau, Ana María Contardo Ayala, Harriet Koorts

**Affiliations:** 1 Institute for Physical Activity and Nutrition (IPAN), School of Exercise and Nutrition Sciences, Deakin University, Geelong, Australia; 2 School of Exercise Science, Physical and Health Education, University of Victoria, Victoria, Canada; 3 Department of Public and Occupational Health and Amsterdam Public Health research institute, Amsterdam UMC, Vrije Universiteit Amsterdam, Amsterdam, The Netherlands

**Keywords:** School, Physical activity, Sedentary behaviour, Implementation, Dissemination, Children, Adolescents, Implementation theory, Implementation models, Implementation frameworks

## Abstract

**Background:**

Globally, many children fail to meet the World Health Organization’s physical activity and sedentary behaviour guidelines. Schools are an ideal setting to intervene, yet despite many interventions in this setting, success when delivered under real-world conditions or at scale is limited. This systematic review aims to i) identify which implementation models are used in school-based physical activity effectiveness, dissemination, and/or implementation trials, and ii) identify factors associated with the adoption, implementation and sustainability of school-based physical activity interventions in real-world settings.

**Methods:**

The review followed PRISMA guidelines and included a systematic search of seven databases from January 1st, 2000 to July 31st, 2018: MEDLINE, EMBASE, CINAHL, SPORTDiscus, PsycINFO, CENTRAL, and ERIC. A forward citation search of included studies using Google Scholar was performed on the 21st of January 2019 including articles published until the end of 2018. Study inclusion criteria: (i) a primary outcome to increase physical activity and/or decrease sedentary behaviour among school-aged children and/or adolescents; (ii) intervention delivery within school settings, (iii) use of implementation models to plan or interpret study results; and (iv) interventions delivered under real-world conditions. Exclusion criteria: (i) efficacy trials; (ii) studies applying or testing school-based physical activity policies, and; (iii) studies targeting special schools or pre-school and/or kindergarten aged children.

**Results:**

27 papers comprising 17 unique interventions were included. Fourteen implementation models (e.g., RE-AIM, Rogers’ Diffusion of Innovations, Precede Proceed model), were applied across 27 papers. Implementation models were mostly used to interpret results (*n* = 9), for planning evaluation and interpreting results (*n* = 8), for planning evaluation (*n* = 6), for intervention design (*n* = 4), or for a combination of designing the intervention and interpreting results (*n* = 3). We identified 269 factors related to barriers (*n* = 93) and facilitators (*n* = 176) for the adoption (*n* = 7 studies), implementation (*n* = 14 studies) and sustainability (*n* = 7 studies) of interventions.

**Conclusions:**

Implementation model use was predominately centered on the interpretation of results and analyses, with few examples of use across all study phases as a planning tool and to understand results. This lack of implementation models applied may explain the limited success of interventions when delivered under real-world conditions or at scale.

**Trial registration:**

PROSPERO (CRD42018099836).

## Contributions to the literature

- Real-world implementation and scale-up of school-based physical activity and sedentary behaviour studies remains uncommon, but critical to achieving population health goals.
- This paper identifies where and how to improve efforts to understand how to enhance adoption, implementation and sustainability of school-based physical activity interventions under real-world conditions which is a necessary ingredient to advancing implementation science in this field and setting.
- Improving the use of theory/model driven approaches and common language across the implementation research spectrum in school-based interventions from planning through to measurement and interpretation is highlighted. This push to include theory driven approaches and to further outline best practices for terminology and reporting is common across disciplines but important to discuss specifically in relation to physical activity interventions.

## Background

Physical inactivity is a worldwide pandemic and leading cause of non-communicable disease [1]. Increased physical activity and decreased sedentary behaviour are associated with positive health impacts and healthy development in children [2, 3], and physical activity provides benefits for school-related outcomes such as classroom behaviour, cognitive function, and academic achievement [4–6]. Nonetheless, the 2018 Global Matrix 3.0 Physical Activity Report Card, which included 49 countries, showed that a minority of school-aged children are meeting internationally recognised guidelines for physical activity (27–33%) by accumulating at least 60 min of moderate- to-vigorous-intensity physical activity daily, and sedentary behaviour (34–39%) which recommend no more than 2 hours of screen time per day [7].

Schools have been proposed as an ideal setting to intervene [8] with numerous calls from the WHO to implement school-wide physical activity promotion programmes [9, 10]. This has led to a number of studies and systematic reviews of efficacy trials which provide evidence of reduction in sedentary behaviour, increased time spent in overall physical activity and in-school physical activity for children exposed to school-based interventions [11, 12]. In a 2013 Cochrane Review, Dobbins et al. showed that increases in physical activity ranged from five to 45 min per day and that television watching, as a marker of sedentary behaviour, was reduced by five to 60 min per day [11]. Despite showing promising findings, this review and others to date, have mostly focused on investigating interventions delivered in controlled settings, or have included studies of school policies rather than interventions, and have not reported on the implementation frameworks, models and theories (‘implementation models’) used to support this evaluation process [13–16]. There has also been far less research describing how interventions are adopted, implemented and sustained under real-world conditions (e.g. implementation studies, or studies which tested the effectiveness, scale-up, dissemination or translation of interventions) [17–20]. By ‘real-world’ we are referring to interventions delivered by school employees during their standard practice in the education system. Real-world interventions require a better understanding of the complex systems in which contextual factors, including organisations, intervention agents (i.e. implementers), target population and setting level social influences (e.g. organisational culture), are typically less controlled than they are efficacy research designs [21]. In this instance, adoption occurs when an organisation (e.g. school) makes a formal decision to commit to using an intervention or policy [22], whereas implementation refers to the processes involved in integrating interventions or policy within organisations and settings [23]. Sustainability relates to the continued use of an intervention with ongoing positive intervention outcomes [24].

Understanding how and what affects the real-world adoption, implementation and sustainability of interventions is critical, as interventions need to be designed for delivery in real-world conditions to have a population-wide impact [18]. We know from a 2015 review by Naylor et al. [14] that the level of implementation is linked to efficacy and outcomes of school-based physical activity interventions. Their review also describes factors that facilitated and hindered implementation based on Durlak and DuPre’s implementation model [25]. An acknowledged limitation in the review is that findings may not be generalisable to real-world systems as they stem predominantly from efficacy trials and more work is needed to assess interventions when interventions are delivered under real-world conditions at scale [14]. Systematic review evidence from obesity prevention research suggests scaled-up interventions are less effective than their initial efficacy trials [26]. The difficulty of achieving intervention effects at scale may, in part, be due to adaptations which are necessary to translate complex interventions originally delivered under controlled circumstances into real-world settings [26]. This may also highlight the level of planning required for effective real-world implementation [27, 28] and the inherent limitations of attempting to translate interventions from highly controlled conditions into ‘real-world settings [29]. Thus, to better understand how to improve the real-world impact of physical activity and sedentary behaviour interventions, there is a need to review the factors associated with adoption, implementation, and sustainability of interventions delivered in real-world settings.

Schools face many challenges in translating evidence-based interventions into routine practice (e.g. funding, school climate, teacher self-efficacy, curriculum demands, and implementation support, among others) [30–32]. Therefore the use of implementation theory is recommended to underpin the processes of planning, implementing and evaluating interventions, especially in the case of complex, multifaceted health promotion programs [33, 34]. To this end, numerous implementation theories, frameworks and models have been developed and collated [33, 34]. Unfortunately, despite the existence of multiple implementation models and appeals for more systematic reviews investigating the application of evidence-based programs in everyday practice [35], there still remains a lack of research, particularly regarding issues of sustained practice [19, 20].Whilst we know ‘why’ implementation models are selected (i.e., empirical support, description of implementation processes, or researcher familiarity) [36], it is unclear ‘how’ they are used in the practice of physical activity school-based prevention research. This review aims to offer important insights into future intervention development and delivery at a population level by: 1) identifying which implementation theories, frameworks, and models (hereafter referred to as “implementation models”) are used in real-world school-based physical activity and/or sedentary behaviour trials; and 2) identifying barriers and facilitators associated with the adoption, implementation and sustainability of interventions in real-world settings.

## Methods

This review was prospectively registered with PROSPERO (CRD42018099836) and follows the Preferred Reporting Items for Systematic Reviews and Meta-Analyses (PRISMA) guidelines [37] (Additional file 5).

### Eligibility criteria

Inclusion criteria were studies which: i) included school-aged children or adolescents; ii) involved interventions delivered in the school setting during school hours with a primary outcome to either increasing physical activity and/or decreasing sedentary behaviour; iii) applied implementation models to plan or to interpret study results; and iv) were conducted in real-world settings (e.g. effectiveness, scale-up, dissemination, translation, and implementation studies). As this review focuses on studies conducted in real-world settings, inclusion of a control group was not a criterion for eligibility. Studies were excluded when they: i) tested efficacy (e.g. randomised controlled trials, feasibility and pilot studies); ii) were conducted with special schools or pre-school and/or kindergarten aged children; and iii) applied or tested school-based physical activity policy (i.e. no program was implemented).

### Information sources and searches

We searched the online databases of MEDLINE, EMBASE, CINAHL, SPORTDiscus, PsycINFO, CENTRAL, and ERIC for peer-reviewed English language articles published on or after January 1st, 2000 until the 31st of July 2018. A research librarian was consulted during the development and testing of search terms (Additional file 1). Reference lists of included studies were hand-searched for eligible interventions and a forward citation search using Google Scholar was performed on the 21st of January 2019 including articles published until the end of 2018.

### Study selection

One author (SC) screened article titles. All abstracts and full texts were screened by two authors (SC and AM) with discrepancies on study inclusion discussed and a consensus agreement made by five authors (SC, AM, HK, JS, and AT). Reference lists and forward searching was undertaken by SC and inclusion decisions were made by consensus agreement by four authors (SC, HK, JS, and AT).

### Data collection process

Data were extracted by one author (SC), with authors (AM, HK, JS, and AT) consulted for clarification where necessary. Data extracted included: date, study population, study design, intervention strategies and location, implementation model use, implementation strategies, implementation measures, factors related to adoption, implementation, sustainability, and results and comments. As the studies included in this review did not all include evidence on the effectiveness of the interventions, we were unable to report the impact of each of the factors described above on overall intervention success. The need to research the relative importance of the factors listed in this review is highlighted for future research.

#### Data synthesis, extraction and quality assessment

Implementation models applied in the included studies (Aim 1) were first grouped within Nilsen’s five categories [33]: (i) process models (used to describe or guide implementation), (ii) determinant frameworks (helpful to understand what influences implementation outcomes), (iii) classic theories, (stemming from fields outside implementation research and used to understand or explain aspects of implementation), (iv) implementation theories (which aim to describe and understand features of implementation), and (v) evaluation frameworks (to guide relevant features of successful implementation). Secondly, for each included study implementation models were characterised per their reported application to either: (i) design the intervention, (ii) plan the evaluation, (iii) interpret the results, or any combination of the three. Factors related to adoption, implementation and sustainability, and barriers and facilitators related to implementation were extracted and grouped (Aim 2). Factors were then categorised according to Durlak and DuPre’s [25] framework, which highlights 23 contextual factors related to the five domains of the delivery system, support system, the providers, aspects of the intervention, and the communities in which they are implemented. Following categorisation, factors were then consolidated, and intervention specific terminology was generalised. All factor categories were discussed among SC, AT, JS and HK before consensus decisions were made on final groupings. Analysing factors within the scope of this framework enabled comparisons of factors between studies, including those found to be relevant in Naylor et al.’s [14] review.

The Mixed Methods Appraisal Tool (MMAT) was used independently by two authors (SC and AM) to assess study quality [38]. The MMAT was developed to enable the assessment of different study designs by offering a single tool consisting of different criteria for quantitative, qualitative and mixed methods studies [39]. The tool includes two screening questions, in addition to five questions per study design in which response options include: yes; no; and can’t tell. For the purposes of this review, questions relating to the qualitative studies, non-randomized studies, quantitative descriptive, and mixed-methods studies were included. Where multiple publications have been published for any one intervention, publications were grouped, and an overall assessment made for the intervention. As overall scores assigned to interventions are discouraged because they do not allow readers to see which aspects of the studies have been covered or not, the MMAT instead recommends users to provide a presentation of the ratings (see Additional file 6). Initial inter-rater reliability was determined using Cohen’s κ, showing moderate agreement 86.1% (κ = 0.56).

## Results

### Study selection

**Fig. 1:**
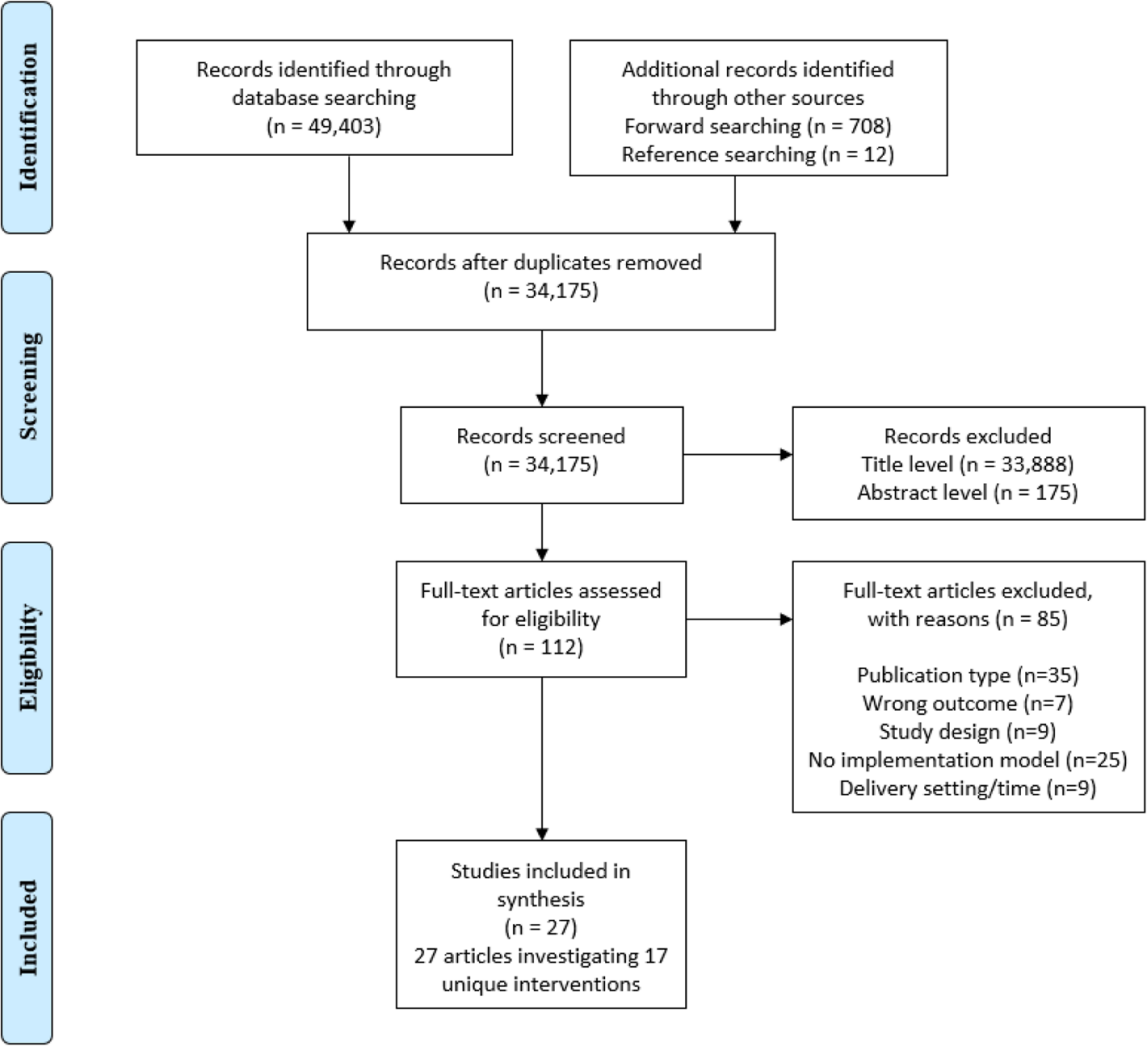
PRISMA flow diagram- Search strategy and inclusion

The study selection and screening process is outlined in Fig. 1. The electronic database search identified 33,445 unique records. An additional 12 records were identified from reference searching and 708 records from our forward searching which resulted in 34,175 records for screening. A total of 33,888 records were excluded at the title level and 175 at the abstract level, thus 112 full texts were assessed for eligibility. Full texts were excluded (*n* = 85) due to publication type (e.g. editorials, commentary papers), outcome other than physical activity/sedentary behaviour, inappropriate study design, absence of implementation model, and inappropriate delivery setting/ time (e.g. outside school hours). Thus, 27 papers, comprising 17 unique interventions were included in this review [40–66].

### Study characteristics

Of the 27 papers included in this review and outlined in Table 1, five employed a qualitative study design [45, 54, 57, 59, 61], nine a quantitative design [42, 44, 48, 50–52, 60, 62, 65], ten utilised mixed-methods [40, 41, 43, 46, 47, 55, 59, 63, 64, 66], and three included summary articles which collated previous findings and discussed lessons learned across multiple publications for a specific intervention [49, 53, 56]. Interventions conducted in the articles were delivered in six high income countries, as categorised by the World Bank [67]: USA [41–44, 48–51, 56, 58, 60, 65, 66], Canada [52, 53, 59], Netherlands [46, 47, 61–64], United Kingdom [45, 57], Australia [40], and Denmark [54, 55]. Further, interventions were conducted in a range of school settings including primary/elementary [40, 42, 44–47, 49, 51, 54–57], middle [43, 48, 66], primary/middle [52, 53, 59], high [58], pre-vocational [61–64], and all ages (primary, middle and high) [41, 50].

**Table 1:** Intervention implementation models and factors associated with adoption, implementation, and sustainability

	Intervention (N = number of studies)	Implementation model (s)*	Adoption factors	Implementation factors	Sustainability factors
1	DOiT *N* = 4 (61–64)	^a, b, c, l, m, n^	✔	✔	✔
2	Action Schools! BC *N* = 2 (52, 53)	^a, c, d, g^		✔	
3	Svendborg project *N* = 2 (54, 55)	^b, d, g, j^	✔	✔	
4	Jump-in! *N* = 2 (46, 47)	^b, f, l^	✔	✔	✔
5	Lifestyle education for activity program (LEAP) *N* = 1 (58)	^d, k^		✔	
6	Child and Adolescent Trial for Cardiovascular Health (CATCH) *N* = 4 (42, 49, 51, 56)	^a^		✔	✔
7	Planet Health *N* = 2 (49, 66)	^a^	✔	✔	✔
8	Fuel Up to Play 60 *N* = 2 (41, 50)	b	✔	✔	✔
9	NFL PLAY 60 FitnessGram® *N* = 1 (65)	^b, f^			
10	Unnamed intervention *N* = 1 (60)	^i^		✔	
11	Marathon Kids *N* = 1 (45)	^a^	✔	✔	
12	Exercise Your Options *N* = 1 (48)	^b^			
13	Students for Nutrition and eXercise (SNaX) *N* = 1 (43)	^b^			
14	The Daily Mile *N* = 1 (57)	^e^		✔	✔
15	Apple Schools *N* = 1 (59)	^h^		✔	✔
16	PLAY (promoting lifelong active youth) Zone (PZ) *N* = 1 (40)	^b^	✔	✔	
17	Structured classroom physical activity programs *N* = 1 (44)	^e^		✔	

*Implementation models represented by the following superscripts: ^a^ Rogers’ Diffusion theory, ^b^ RE-AIM, ^c^ Multilevel implementation quality framework, ^d^ Ecological framework for understanding effective implementation, ^e^ Consolidated Framework for Implementation Research (CFIR), ^f^ Precede Proceed model, ^g^ A Conceptual Framework for Implementation, ^h^ Conceptual Model of School-Based Implementation, ^i^ Ambiguity-conflict model of policy implementation, ^j^ What Does It Take? Implementation of evidence-based programs, ^k^ Measuring persistence of implementation, ^l^ Determinants of innovation within health care organizations, ^m^ Process Evaluation for Public Health Interventions and Research, ^n^ Process Evaluation Plan of Saunders et al.

Quality assessment scores have been reported in Additional file 6. Briefly, the three qualitative studies all scored a ‘yes’ for the seven items. The quantitative studies were of lower quality comparatively, with four of the six studies receiving a ‘no’ for the item ‘Is the risk of nonresponse bias low?’, with one ‘can’t tell’ and one ‘no’ for the item ‘Is the sample representative of the target population?’. Of the eight mixed-methods studies, two scored a ‘yes’ for all of the 17 related items. For the other six studies, items relating to qualitative aspects were least likely to receive a ‘yes’, with items ‘Are the qualitative data collection methods adequate to address the research question?’ and ‘Is there coherence between qualitative data sources, collection, analysis and interpretation?’ both receiving four ‘can’t tell’ responses.

### Implementation model application

**Fig. 2:**
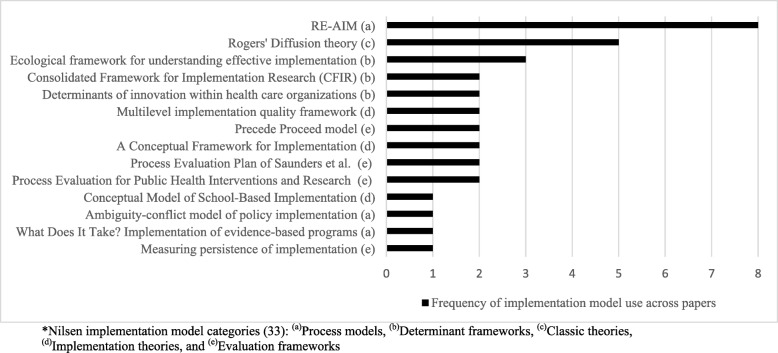
Frequency of implementation model use per intervention

Fourteen implementation models were applied 34 times in the 27 included papers (Fig. 2). Eight implementation models were utilised by at least two separate interventions, including: RE-AIM [21], Rogers’ Diffusion of Innovations theory [22], Ecological framework for understanding effective implementation [25], Consolidated Framework for Implementation Research (CFIR) [68], Determinants of innovation within health care organizations [69], Multilevel implementation quality framework [30], Precede Proceed model [70], and A Conceptual Framework for Implementation [71]. Of the 14 implementation models applied in the included studies, all five of Nilsen’s [33] categories; Evaluation frameworks (*n* = 5), Implementation theories (*n* = 3), Determinant frameworks (*n* = 3), Process models (*n* = 2) and Classic theories (*n* = 1) were represented, underlining the variety of models used in the field.

The most common use of implementation models across studies were to interpret results (*n* = 9), followed by a combination of planning the evaluation and interpreting the results (*n* = 8). Implementation models were also used to plan the evaluation (*n* = 6), solely in the design of the intervention (*n* = 4), to design the intervention and interpret results (*n* = 3), to design the intervention and plan the evaluation (*n =* 1) and finally in a combination of all three aspects to design the intervention, plan the evaluation and interpret the results (*n* = 1).

### Barriers and facilitators in intervention adoption, implementation and sustainability phases

Of the included papers reviewed, seven described factors pertinent to adoption, 14 considered aspects related to implementation, and seven discussed influences on sustainability. A total of 275 factors were reported across the three phases, with 52 factors related to adoption (facilitators *n* = 36, barriers *n* = 16), 154 factors linked to implementation (facilitators *n* = 107, barriers *n* = 47), and 63 factors linked to sustainability (facilitators *n* = 33, barriers *n* = 30). A full list of these factors are organised under the five domains relating to the Durlak and DuPre model [25]: community level factors; provider characteristics; characteristics of the innovation; factors relevant to the delivery system; and factors related to the prevention support system (See Additional file 2, Additional file 3, and Additional file 4).

Table 2 highlights the domains covered for each individual phase of adoption, implementation, and sustainability to illustrate the impact (barriers/facilitators) and coverage of factors across the dissemination continuum. The following section contains a list outlining the category groups covered for each phase with examples in parentheses taken from included articles. In total, there were seven category groups reported as a facilitator for all three phases of adoption, implementation and sustainability: 1) Policy (e.g. Aligned with state education standard); 2) Perceived benefits of innovation (e.g. Classroom behaviour benefits); 3) Compatibility (e.g. Feasible and acceptable); 4) Adaptability (e.g. Flexible approach to commencing implementation); 5) Integration of new programming (e.g. Easy to integrate in organisations); 6) Coordination with other agencies (e.g. Willingness/aptitude to collaborate); and 7) Managerial support (e.g. Teachers encouraged/ supported by school to trial intervention).

**Table 2:** Durlak and DuPre domains covered by each dissemination phase

Durlak and DuPre domains	Adoption		Implementation		Sustainability	
	Barriers	Facilitators	Barriers	Facilitators	Barriers	Facilitators
Community Level Factors	✓	✓		✓	✓	✓
Prevention Theory and Research	✓			✓	✓	
Politics						✓
Funding				✓	✓	✓
Policy	✓	✓		✓	✓	✓
Provider Characteristics	✓	✓	✓	✓	✓	✓
Perceived Need for Innovation	✓	✓	✓		✓	✓
Perceived Benefits of Innovation		✓	✓	✓	✓	✓
Self-efficacy				✓		
Skill Proficiency				✓	✓	
Characteristics of the Innovation	✓	✓	✓	✓	✓	✓
Compatibility	✓	✓	✓	✓	✓	✓
Adaptability		✓	✓	✓		✓
Availability/Quality of resources^a^		✓				
Factors Relevant to the Prevention Delivery System: Organizational Capacity	✓	✓	✓	✓	✓	✓
General Organizational Factors		✓				
Positive Work Climate				✓		
Organizational norms regarding change				✓		
Integration of new programming	✓	✓	✓	✓	✓	✓
Shared vision				✓		✓
Shared decision-making		✓		✓		
Coordination with other agencies		✓	✓	✓	✓	✓
Communication	✓	✓	✓	✓		
Formulation of tasks			✓	✓	✓	✓
Specific Staffing Considerations	✓		✓		✓	
Leadership	✓		✓		✓	✓
Program champion			✓	✓		
Managerial/supervisory/administrative support		✓	✓	✓		✓
Characteristics of the school^a^	✓	✓	✓	✓		
Classroom management/ Disruptive student behaviour^a^						✓
Factors Related to the Prevention Support System		✓	✓	✓	✓	✓
Training			✓	✓		✓
Technical Assistance		✓		✓	✓	
Others^a^		✓	✓			✓
Student characteristics, engagement and motivation^a^		✓				✓
Parent support and perceptions			✓			

^a^Other categories as per the classification proposed by Naylor et al. [14]

Correspondingly, five category groups were reported as barriers across each phase: 1) Perceived need for innovation (e.g. Low priority relative to other academic subjects); 2) Compatibility (e.g. Program too complex for education level); 3) Integration of new programming (e.g. Need for simplified methods, instruments, protocols, and tasks); 4) Specific staffing considerations (e.g. Teacher attrition); and 5) Leadership (e.g. Change in school leadership). Aspects related to the compatibility and integration of new programming were the only two category groups to be listed as facilitators and barriers across all three phases. Further, several category groups were listed in at least two of the phases, with the majority of these listed as facilitators stemming from factors relevant to the delivery system (schools’ organisational capacity) and the prevention support system. A full list of facilitators and barriers relating to the adoption, implementation and sustainability are reported in Additional file 2, Additional file 3, and Additional file 4.

### Adoption

Facilitating factors specifically related to adoption were identified across 16 category groups. Facilitators relevant to domains ‘characteristics of the innovation’ (*n* = 13) and ‘the prevention delivery system’ (*n* = 15) were presented most frequently. Adoption barriers were reported in the following nine category groups. Factors related to ‘the prevention delivery system’ (*n* = 9) were barriers represented the most frequently.

### Implementation

Implementation facilitators were reported across all five domains and comprised 21 category groups. Factors relating to ‘the prevention delivery system’ (*n* = 42) were represented most frequently. Implementation barriers were mentioned across all domains with the exception of community level factors, covering a total of 15 different categories. Of which, factors relevant to the ‘prevention delivery system’ (*n* = 39) were most frequently reported.

### Sustainability

Facilitators for the sustainability of school-based interventions were reported across all five domains and included factors from 16 category groups. Sustainability barriers again covered all five domain headings across 14 category groups. Factors under ‘the prevention delivery system’ domain were the most prevalent for both sustainability facilitators (*n* = 12) and barriers (*n* = 18).

## Discussion

This review assessed the use of implementation models in 17 school-based interventions aiming to increase physical activity and/or reduce sedentary behaviour interventions implemented under real-world conditions, and identified facilitators and barriers associated with the adoption, implementation and sustainability of these interventions. The review contributes to the existing evidence base by identifying and comparing factors relevant to implementation under largely uncontrolled conditions and mapping them against a well-recognised implementation framework [25] to identify patterns that will move implementation research on school-based physical activity interventions forward. However, we faced difficulties with comparing identified factors and themes because of the variability in use of terminology across implementation research, previously described as a ‘Tower of Babel’ [72]. Thus it is important for future studies to clearly and systematically label intervention strategies and outcomes [73–76], and to follow recommended reporting mechanisms such as the purpose designed Standards for Reporting Implementation Studies (STARI) statement [77].

In reviewing facilitators and barriers for real-world physical activity and sedentary behaviour interventions in schools, we encountered a broader evidence base for factors which influence the implementation phase (such as implementation support strategies and implementation fidelity), in comparison to literature discussing influences pertinent to the adoption or sustainability of interventions. Further research on factors associated with adoption and sustainability of interventions is warranted given that previous studies show barriers and facilitators differ across phases [19, 25, 30, 31].

### The application of implementation models in school-based intervention studies

In total, 14 different implementation models were applied across interventions, with eight applied on more than two occasions and three (RE-AIM [21], Roger’s diffusion theory [22], and Ecological framework for understanding effective implementation [25] standing out as most often utilised. The most common use of an implementation model was predominately centred around the interpretation of results and analyses, with few examples of studies which applied implementation models as a tool across all phases of the study (e.g. as a planning tool for intervention components, as a tool to evaluate the intervention effect and as a tool to interpret study results/findings). This is underlined by the Nilsen [33] groupings, as implementation models under the category of ‘Evaluation frameworks’ were most commonly cited across studies. The unsystematic application of implementation models at different phases, and in some cases in a retrospective manner, precludes their applicability as a guiding tool throughout the entire intervention process, and may contribute to limitations in the field’s understanding of key mechanisms and phases [34]. Our findings are in line with a previous systematic review of studies citing the Consolidated Framework for Implementation Research (CFIR) [78] that found more than 80% of studies did not apply the model in a meaningful manner (i.e. CFIR was not used to guide the methodology of study design, analysis or interpretation of results) [79]. Their review also highlighted that more than half of the included studies used the implementation model for data analysis purposes and further, that only 23% of studies applied the framework to both guide data collection and analysis. The authors report that using an implementation model was advantageous as a checklist in guiding data collection and ensured that important unmeasured factors were not uncovered during data analysis [79]. The selective and sporadic application of implementation models in their review appear to mirror our findings and alludes to the seemingly ad hoc application of models in implementation research also noted in the implementation literature [36, 73]. In recognition of the under- and ad hoc utilisation of implementation models, and the understanding that researchers may need support in the selection and application of implementation models [80], a number of publications [34, 36, 81] and tools [82–84] have been developed which aim to guide this process. For researchers and practitioners seeking to plan clinical and community interventions implemented at scale, the previously mentioned PRACTical planning for Implementation and Scale-up (PRACTIS) guide represents another example of recent work providing practical direction [28].

### Barriers and facilitators to adoption, implementation and sustainability

Despite these potential differences across phases, our review suggests that several barriers and facilitators, in particular factors relating to intervention compatibility and the integration of new programming, remained common across the three phases of adoption, implementation, and sustainability (See Table 2). We report on these category groups here as they represent action areas which may prove to be a list of ‘best buys’ for intervention planning and development.

Across all three phases of adoption, implementation and sustainability, factors relating to the school ‘Delivery system’ were most often cited as facilitators and barriers. This implies the importance of schools and change agents (including researchers) addressing these barriers through organisational policies and practices which support the delivery of new interventions. We encourage schools and change agents wanting to adopt, implement and sustain new interventions to consider how they can best prepare their staff when introducing new interventions. In particular schools and intervention developers should work together to limit the impact of anticipated barriers and to harness the benefits of identified facilitators.

One such way to increase the likelihood of implementation of interventions in everyday practice, includes utilising tools such as the PRACTIS guide which encourages early planning for anticipated barriers at the individual, organisational and systems levels [28]. These barriers can then be linked to implementation strategies which best address the specific contextual determinants of implementation [85]. School-level, organisational factors reported above which include managerial support, coordination with other agencies, and specific staffing considerations are a key determinant of successful implementation and have been described as such both within and outside of the education sector [86–89]. Perceptions regarding the need for and benefits of the intervention also seem central, as well as the compatibility and adaptability of programs, thus supporting Rogers’ seminal diffusion of innovation model [22] among others [25, 30, 78]. For example, designing interventions which involve changes to the pedagogical style (e.g. active lessons) rather than changes in curriculum may be a useful strategy moving forward. Additionally, it seems pertinent to focus on the language used to promote the need and benefits of these intervention using school-related (i.e. improvements in classroom focus and improved academic performance) rather than the traditional approach of highlighting the impact of physical inactivity on health.

Despite several factors being relevant across the dissemination continuum, our review found various phase-specific factors and therefore supports recommendations put forward in the Conceptual Model of School-Based Implementation that implementation strategies need to be tailored for each phase [30]. This suggests schools, researchers and change agents should consider strategies utilised during the adoption phase are not necessarily the same needed during the implementation phase and further, that to ensure sustainability, a separate set of conditions and factors may be relevant [31].

## Limitations

Major strengths of this review include the application the Durlak & DuPre model [25], an established implementation model to enable the comparison of facilitators and barriers across other reviews [14]. Secondly, our review demonstrates the diversity in application of implementation models in real-world trials across the three phases of the dissemination continuum. However, this review is not without limitations. Firstly, there were perhaps other interventions that have been implemented under real-world conditions that have collated factors relevant to adoption, implementation, or sustainability which are not included in this review because they didn’t meet the inclusion criteria of ‘using an implementation model’. This is therefore not an exhaustive list of all factors relevant to adoption, implementation and sustainability of real-world interventions. Papers rarely reported separately on implementation of physical activity and sedentary behaviours and it is certainly possible that barriers and facilitators to implementation could differ. We further note the absence of studies stemming from low- and middle-income countries, and suggest further research is needed to complement our findings and expand the literature base regarding issues faced in these countries. Results discussing use of models may not capture full application of the model as use of the model was simply extracted from the authors’ description and there may be instances where it was inferred that one use automatically led to its’ application in another form. Finally, the identified facilitators and barriers may not necessarily be ‘significant’ or result in meaningful changes in effectiveness and may share the same name but have been measured in a different way (e.g. qualitative interviews or focus groups vs quantitative surveys, or different definition of variables).

## Conclusions

Our review highlights the selective and sporadic application of implementation model components and alludes to a seemingly ad hoc application which focuses more so on the interpretation of results than of a holistic application across the lifespan of an intervention (i.e. designing the intervention, planning the evaluation, and interpreting the results). Further, this study reviews the growing literature describing school-based physical activity interventions conducted under real-world conditions by mapping factors related to the adoption, implementation and sustainability against a recognised implementation model. The key message for practice being that the application of implementation models from intervention inception can aid researchers and practitioners to leverage known facilitators and mitigate the impact of barriers. Finally, further research is needed, particularly during the adoption and sustainability phases, to assist in the development of strategies which facilitate the process of implementing school-based physical activity interventions in real-world conditions at scale.

## Supplementary information

**Additional file 1.** Search terms and databases.

**Additional file 2.** Factors related to the adoption of real-world school-based interventions.

**Additional file 3.** Factors related to the implementation of real-world, school-based interventions.

**Additional file 4.** Factors related to the sustainability of real-world, school-based interventions.

**Additional file 5.** PRISMA checklist.

**Additional file 6.** MMAT risk of bias tool.

## Data Availability

N/A.

## Abbreviations

CFIR: Consolidated Framework for Implementation Research
PRACTIS: PRACTical planning for Implementation and Scale-up
STARI: Standards for Reporting Implementation Studies
WHO: World Health Organization
