# Supplementary material for: Adoption, implementation and sustainability of school-based physical activity and sedentary behaviour interventions in real-world settings: a systematic review
Source: Int J Behav Nutr Phys Act. 2019 Dec 2;16:120. doi: 10.1186/s12966-019-0876-4 (PMC6889569; doi:10.1186/s12966-019-0876-4)
Supplement: Supplementary file 1 — Additional file 1. Search terms and databases. [file 12966_2019_876_MOESM1_ESM.docx]

Additional file 1. Search terms and databases

MEDLINE Complete

S43 S29 AND S32 AND S42

S42 S33 OR S34 OR S35 OR S36 OR S37 OR S38 OR S39 OR S40 OR S41

S41 AB ( (translation* or dissemination or implementation) N2 (framework* or model*or plan* or approach*or strateg* or protocol* or guideline* or manual* or concept*) ) OR TI ( (translation* or dissemination or implementation) N2 (framework* or model*or plan* or approach*or strateg* or protocol* or guideline* or manual* or concept*) )

S40 AB ( (monitor* or measur*) N5 implementation ) OR TI ( (monitor* or measur*) N5 implementation )

S39 AB process N5 evaluation OR TI process N5 evaluation

S38 AB ( (program* or intervention) N5 (delivery or uptake or adopt*or adapt* or modif*) ) OR TI ( (program* or intervention) N5 (delivery or uptake or adopt*or adapt* or modif*) )

S37 AB ( adopt* or uptake or utilisation or utilization or sustain* or program* or initiative* or feasib* ) OR TI ( adopt* or uptake or utilisation or utilization or sustain* or program* or initiative* or feasib* )

S36 AB ( “scale up” or “scaled up” or “scaling up” or scaling or scalability or “scale out” or translat* or “roll out” or “rolled out” or “real-world” or “research to practice” ) OR TI ( “scale up” or “scaled up” or “scaling up” or scaling or scalability or “scale out” or translat* or “roll out” or “rolled out” or “real-world” or “research to practice” )

S35 AB ( implement* or disseminat* or diffus* ) OR TI ( implement* or disseminat* or diffus* )

S34 (MH “Evaluation Studies”)

S33 (MH “Program Evaluation”)

S32 S30 OR S31

S31 AB ( school or “secondary college” or “school-based” or “primary education” or “secondary education” or école or academy or gymnasium ) OR TI ( school or “secondary college” or “school-based” or “primary education” or “secondary education” or école or academy or gymnasium )

S30 (MH “Schools”)

S29 S1 OR S2 OR S3 OR S4 OR S5 OR S6 OR S7 OR S8 OR S9 OR S10 OR S11 OR S12 OR S13 OR S14 OR S15 OR S16 OR S17 OR S18 OR S19 OR S20 OR S21 OR S22 OR S23 OR S24 OR S25 OR S26 OR S27 OR S28

S28 AB ( lifestyle N5 (physical* or activ*) ) OR TI ( lifestyle N5 (physical* or activ*) )

S27 AB walk* OR TI walk*

S26 AB sport* OR TI sport*

S25 AB ( exercis* N5 (train* or physical* or activ*) ) OR TI ( exercis* N5 (train* or physical* or activ*) )

S24 AB strength* N5 train* OR TI strength* N5 train*

S23 AB ( “active trans*” or “active trav*” or “active comm*” ) OR TI ( “active trans*” or “active trav*” or “active comm*” )

S22 AB ( physical* N5 (fit* or train* or activ* or endur*) ) OR TI ( physical* N5 (fit* or train* or activ* or endur*) )

S21 AB exercise* N5 aerobic* OR TI exercise* N5 aerobic*

S20 AB dancing OR TI dancing

S19 (MH “dancing”)

S18 (MH “sports”)

S17 (MH “walking”)

S16 (MH “leisure activities”)

S15 (MH “physical fitness“)

S14 (MH “Physical Education and Training”)

S13 (MH “motor activity”)

S12 (MH “exercise”)

S11 AB ( “fundamental movement skills” or “fundamental motor skills” or FMS ) OR TI ( “fundamental movement skills” or “fundamental motor skills” or FMS )

S10 AB “physic* activ*” OR TI “physic* activ*”

S9 AB “physical* inactiv*” OR TI “physical* inactiv*”

S8 AB ( physical* N2 (inactivit* or “insufficient* activ*” or inactive or “inadequat* active*”) ) OR TI ( physical* N2 (inactivit* or “insufficient* activ*” or inactive or “inadequat* active*”) )

S7 AB sitting OR TI sitting

S6 AB ( smartphone* or “smart phone*” or “cell phone*” or “mobile phone*” or “small screen*” or iphone* or ipad* or ipod* or tablet* or laptop* or computer or “handheld device” or “screen based*” or “screen time” ) OR TI ( smartphone* or “smart phone*” or “cell phone*” or “mobile phone*” or “small screen*” or iphone* or ipad* or ipod* or tablet* or laptop* or computer or “handheld device” or “screen based*” or “screen time” )

S5 AB ( “computer game*” or “video game*” or television or tv or "electronic game*” or gaming or “electronic media” ) OR TI ( “computer game*” or “video game*” or television or tv or "electronic game*” or gaming or “electronic media” )

S4 AB “low energy expenditure” OR TI “low energy expenditure”

S3 AB ( chair or car or automobile or auto or bus or indoor or screen or computer N time ) OR TI ( chair or car or automobile or auto or bus or indoor or screen or computer N time )

S2 AB ( sedentar* or “sed* time” ) OR TI ( sedentar* or “sed* time” )

S1 (MH "sedentary lifestyle")
