# Supplementary material for: Adoption, implementation and sustainability of school-based physical activity and sedentary behaviour interventions in real-world settings: a systematic review
Source: Int J Behav Nutr Phys Act. 2019 Dec 2;16:120. doi: 10.1186/s12966-019-0876-4 (PMC6889569; doi:10.1186/s12966-019-0876-4)
Supplement: Supplementary file 2 — Additional file 2. Factors related to the adoption of real-world school-based interventions. [file 12966_2019_876_MOESM2_ESM.docx]

Additional file 2. Factors related to the adoption of real-world school-based interventions

| **Factors related to adoption** | |
| --- | --- |
| **Facilitators** | **Barriers** |
| **I. Community Level Factors**  *Policy*  -Political advocacy/support^7^ | **I. Community Level Factors**  *Prevention Theory and Research*  -Lack of evidence-based treatment programs for overweight/obese children^7^  *Policy*  -Lack of policy regarding groups at risk^7^ |
| **II. Provider Characteristics**  *Perceived Need for Innovation*  -Program addressing behavioural problems during school breaks^19^  *Perceived Benefits of Innovation*  -Positive outcome beliefs^7^  -Enables greater attention to students with difficulties^6^ | **II. Provider Characteristics**  *Perceived Need for Innovation*  -Lack of perceived need^1^  -Moderate perceived importance of parental information/ extra care by school staff^7^ |
| **III. Characteristics of the Innovation**  *Compatibility*  -Compatible with the school’s values, ambitions, policies and needs^16^  -High perceived fit^7^  -Strong commitment/motivation to comply with shared goals^7^  -Feasibility and compatibility of the programme^1^  -Perceived importance of obesity prevention in the school^1^  -Aligned with this school’s goals^14^  -High perceived importance of goals by director/PE teachers^7^  -Ease of application^19^  *Adaptability*  -Adaptability to offer additional opportunities as part of the school day/outside of PE lessons^16^  -Pupils exercise autonomy over the frequency, duration/mode of participation^16^  -Flexible approach to commencing implementation^12^  *Availability/quality of resources***^#^**  -Packaged program^19^  -Adolescents’ perception: attractiveness of the layout^1^ | **III. Characteristics of the Innovation**  *Compatibility*  -Confusion regarding alignment with district-level requirements^14^  -Complexity of multilevel program causing delay in the preparation period^7^ |
| **IV. Factors Relevant to the Prevention Delivery System: Organizational Capacity**  *General Organizational Factors*  -Incentive Funding^19^  -Availability of financial resources^7^  *Integration of new programming*  -Fits into existing health promotion activities in the school^1^  *Shared vision*  -Strong commitment of teachers/school management^1^  -Teacher: Strong motivation^1^  -Staff buy-in and engagement^16^  *Shared decision-making*  -Shared decision making^1^  -Involvement of experts in tailoring the program to the school^7^  -Co-involvement in the early stages^6^  *Coordination with other agencies*  -Willingness/aptitude to collaborate^7^  -Involvement/support from local health promotion professionals^1^  *Communication*  -Clear information/communication strategies within organisations^7^  *Managerial/supervisory/administrative support*  - Staff support^19^  -Support from senior management/staff buy-in^16^  *Characteristics of the school***^#^**  - Underutilised infrastructure^19^ | **IV. Factors Relevant to the Prevention Delivery System: Organizational Capacity**  *Integration of new programming*  -Feasibility to incorporate ‘extra’ programme into curriculum^1^  -Lack of existing local youth sports clubs^7^  *Communication*  -Unclear internal communication about workload for teachers^1^  *Specific Staffing Considerations*  -Insufficient capacity of local health care workers^7^  -Lack of qualified trainers for lessons with children^7^  -Staff shortages and reassignments^14^  -Changes in their workload/scheduling requirements^14^  *Leadership*  -Top-down decision making, lack of commitment of teachers^1^  *Characteristics of the school***^#^**  -Limited gym space and availability^14^ |
| **V. Factors Related to the Prevention Support System**  *Technical Assistance*  -Availability of website with a toolkit^1^  -Face-to-face information meeting with intervention employee^1^ | **V. Factors Related to the Prevention Support System** |
| **Others^#^**  *Student characteristics, engagement and motivation*  -Student buy-in^19^ | **Others^#^** |

*Studies represented by the following superscripts: ^1^(van Nassau et al. 2016a), ^2^(van Nassau et al. 2016b), ^3^(Mâsse et al. 2012), ^4^(McKay et al. 2015), ^5^(Nielsen et al. 2018a), ^6^(Nielsen et al. 2018b), ^7^(de Meij et al. 2013), ^8^(Saunders et al. 2011), ^9^(Bice, Brown & Parry 2014), ^10^(Franks et al. 2007), ^11^(Hoelscher et al. 2004), ^12^(Wiecha et al. 2004), ^13^(Graziose et al. 2017), ^14^(Beck, Jensen & Hill 2015), ^15^(Totura et al. 2015), ^16^(Chalkley et al. 2018), ^17^(Ryde et al. 2018), ^18^(Storey et al. 2011), ^19^(Austin et al. 2011), ^20^(Carlson et al. 2017).^#^Other categories as per the classification proposed by Naylor et al (14).
