# Supplementary material for: Adoption, implementation and sustainability of school-based physical activity and sedentary behaviour interventions in real-world settings: a systematic review
Source: Int J Behav Nutr Phys Act. 2019 Dec 2;16:120. doi: 10.1186/s12966-019-0876-4 (PMC6889569; doi:10.1186/s12966-019-0876-4)
Supplement: Supplementary file 3 — Additional file 3. Factors related to the implementation of real-world, school-based interventions. [file 12966_2019_876_MOESM3_ESM.docx]

Additional file 3. Factors related to the implementation of real-world, school-based interventions

| **Factors related to implementation** | |
| --- | --- |
| **Facilitators** | **Barriers** |
| **I. Community Level Factors**  *Prevention Theory and Research*  -Planning for diffusion/sustainability from the beginning^10^  -Detection of children with overweight, inactivity, and motor problems succeeded by monitoring system^7^  -Effective identification of children at risk^7^  *Funding*  -Teacher stipends for attendance at the trainings^12^  -Grant funding^13^  *Policy*  -Prepared lessons aligned with state education standard^10^  -Environmental influence (e.g., compliance with Provincial guidelines)^4^  -Attitude, subjective norm/beliefs among political stakeholders^7^ | **I. Community Level Factors** |
| **II. Provider Characteristics**  *Perceived Benefits of Innovation*  -Perceived benefits to implementing classroom physical activity^20^  -Classroom behaviour benefits^20^  -Attitude toward adaptations/changes in policies and practices^7^  -Observable results and perceived advantages^7^  -Outcome beliefs, feasibility, and perceived importance of participants^7^  -Perceived relative advantage of the intervention^10,4^  -Perceived enthusiasm of adolescents^1^  -Outcome expectation^4,5^  *Self-efficacy*  -Self-efficacy^4,5^  -Easy to implement^19^  *Skill Proficiency*  -Percentage of population with postsecondary education^5^  -Teaching experience^5^ | **II. Provider Characteristics**  *Perceived Need for Innovation*  -Perceived responsibility for parental information of school participants^7^  *Perceived Benefits of Innovation*  -Perceived barriers to implementing classroom physical activity^20^  -Lack of practical applications^1^  -Lack of incentives for adolescents^1^ |
| **III. Characteristics of the Innovation**  *Compatibility*  -Fitting the school context^19^  -Feasibility and compatibility of tasks with the regular task orientation^7^  -Aesthetically appealing^19^  -Intervention’s appeal to students^19^  -Well-defined program components^7^  -Materials resonated with the interests/resources of stakeholders^10^  -Layout and content of the materials^1^  -Extensive teacher manual to support implementation^1^  -Intervention simplicity^17^  -Low cost^10^  -Feasible and acceptable^12^  -Existing PE strategies to promote physical activity^16^  -Existing school wellness programs^13^  -Informative and sufficient website^1^  -Quality and frequency of information regarding sports^7^  *Adaptability*  -Adaptability^17^  -High perceived flexibility^1,10,16,17,18^  -Potential for tailoring^1,7^ | **III. Characteristics of the Innovation**  *Compatibility*  -Intervention material perceived too valuable to take home^7^  -Programme too complex for education level^1^  *Adaptability*  -Unbundled workbook, difficulties in copying the separate worksheets^1^ |
| **IV. Factors Relevant to the Prevention Delivery System: Organizational Capacity**  *Positive Work Climate*  -Organisational climate/support^4,5^  -Administrator climate^20^  *Organizational norms regarding change*  -School celebration assemblies^16^  -Launch event^19^  -Games and activities^19^  -Environmental influences^3^  *Integration of new programming*  -Easy to integrate in the lessons for younger groups^7^  -Compatibility to the regular biology, health education^1^  -Level of institutionalisation^4,5^  -Easy to integrate in organisations^7^  *Shared vision*  -Strong commitment/motivation to achieve goals among PE teachers/sport coordinators^7^  -Strong motivation/commitment to work with the programme^1^  -Sufficient collaboration between implementing teachers ^1^  -Organisational commitment/motivation to comply to shared goals^7^  -Visibility of the brand was important for school identity^18^  *Shared decision-making*  -Implementers included in planning and design^5,10^  -Involvement of students^5^  -Participation of all parties was crucial to developing materials^10^  *Coordination with other agencies*  -Clear protocols, tasks, and agreements among organisations^7^  -Clear hierarchical structures within organisations^7^  -Involvement and support of experts in sports, health, and education^7^  -Having intervention staff in the school^5^  -Multidisciplinary character of programme increased collaboration^1^  -Overall coordination of coordinator^1^  *Communication*  -Effective communication strategies between partners in sports^7^  -Close dialogue with programme managers^5^  -Clear/short communication between teachers, sections and teams^1^  *Formulation of tasks*  -Students as peer-support for one-another^16^  -Clear school-wide plan for implementation^1^  -Completing a School Wellness Investigation^13^  *Specific Staffing Considerations*  *Program champion*  -Program champion^16^  -Program promoters^5^  *Managerial/supervisory/administrative support*  -School management commitment^6,19^  -Supportive involvement of a school principal or administrator^10,18^  -Teachers encouraged/supported by school to trial intervention^17^  -Encouraged to hold classroom physical activity^20^  *Characteristics of the school***^#^**  -Availability of physical space^19^  -Schools with >50% students eligible for free/reduced-price lunch^13^  -Schools with more children signed up to the intervention^13^ | **IV. Factors Relevant to the Prevention Delivery System: Organizational Capacity**  *Integration of new programming*  -Incompatibility with existing health care monitoring instruments^7^  -Difficult to integrate in lessons for older groups^7^  -Competing priorities^3,12,20^  -Other priorities^1^  -Short planning time^12^  -Large variation in time teachers spent on the in-class interventions^7^  -Time ^3,20^  -Teacher workload (time)^1^  -Time pressure because of the timeline of the evaluation study^7^  *Coordination with other agencies*  -Complex hierarchical lines/financial structures between collaborating organisations^7^  -Complexity of collaboration between teachers, sections and teams^1^  -Teacher confusion regarding role of intervention employee within the school^3^  -Difficulties to involve parents^1^  -Lack of coordination hampered communication between organisations^7^  -The competing position of some care providers within one district^7^  *Communication*  -Lack of clearness about tasks^7^  -Incorrect indication of time needed for the lessons^1^  *Formulation of tasks*  -Need for more time to fine-tune organisational procedures^7^  -Information/clearness about tasks among the school staff^7^  -Changes in planning for implementation^1^  -A lack of valid and reliable screening instruments to detect children^7^  *Specific Staffing Considerations*  -Teacher attrition due to transfers, retirements, career changes^1,8,12^  -Lack of staff members^19^  -Turnover among politicians, sports coordinators, and PE teachers^7^  -Capacity of staff members^16^  -School directors protect the staff against overload^7^  -Perceived workload among school staff ^7^  -Referrals of overweight/obese children hampered by capacity issues^7^  *Leadership*  -Programme coordinator who is not implementing the programme^1^  *Program champion*  -Champion leaving^19^  *Managerial/supervisory/administrative support*  -Management buy-in^19^  *Characteristics of the school***^#^**  -School closings^12^  -School system attributes^12^  -Availability of gymnasia due to insurance/cleaning agreements^7^  -Space available^16^  -Lack of availability of materials and facilities^1^ |
| **V. Factors Related to the Prevention Support System**  *Training*  -Training^4,5,10,18^  -Tailored professional training/instructions for implementers^7^  -Trained to hold classroom physical activity^20^  -PE teachers participated in professional development^5^  *Technical Assistance*  -Support from the intervention team/a centralised help desk^1,7^  -Availability of intervention staff for training/technical assistance^12^  -Program staff who contact decision makers, answer questions, present at professional meetings, conduct training, ensure quality control^10^  -School- based coordinators to organise interdisciplinary planning^12^  -Availability of resources for classroom physical activity^20^  -Received materials for classroom physical activity^19,20^  -Access to technical assistance classroom physical activity^20^  -External support^19^  -Programme resources provided^16^ | **V. Factors Related to the Prevention Support System**  *Training*  -Teacher professional development^19^ |
| **Others^#^** | **Others^#^**  *Parent support and perceptions*  -Engagement of parents and families^12^  -Difficulties in reaching parents for information meetings and workshops^7^ |

*Studies represented by the following superscripts: ^1^(van Nassau et al. 2016a), ^2^(van Nassau et al. 2016b), ^3^(Mâsse et al. 2012), ^4^(McKay et al. 2015), ^5^(Nielsen et al. 2018a), ^6^(Nielsen et al. 2018b), ^7^(de Meij et al. 2013), ^8^(Saunders et al. 2011), ^9^(Bice, Brown & Parry 2014), ^10^(Franks et al. 2007), ^11^(Hoelscher et al. 2004), ^12^(Wiecha et al. 2004), ^13^(Graziose et al. 2017), ^14^(Beck, Jensen & Hill 2015), ^15^(Totura et al. 2015), ^16^(Chalkley et al. 2018), ^17^(Ryde et al. 2018), ^18^(Storey et al. 2011), ^19^(Austin et al. 2011), ^20^(Carlson et al. 2017).^#^Other categories as per the classification proposed by Naylor et al, (14).
