# Supplementary material for: Adoption, implementation and sustainability of school-based physical activity and sedentary behaviour interventions in real-world settings: a systematic review
Source: Int J Behav Nutr Phys Act. 2019 Dec 2;16:120. doi: 10.1186/s12966-019-0876-4 (PMC6889569; doi:10.1186/s12966-019-0876-4)
Supplement: Supplementary file 4 — Additional file 4. Factors related to the sustainability of real-world, school-based interventions. [file 12966_2019_876_MOESM4_ESM.docx]

Additional file 4. Factors related to the sustainability of real-world, school-based interventions

| **Factors related to Sustainability** | |
| --- | --- |
| **Facilitators** | **Barriers** |
| **I. Community Level Factors**  *Politics*  -Widespread dissemination of the intervention^7^  -National attention^7^  *Funding*  -Structural finances for the intervention^7^  -Funding^12^  -Financial resources; school budget or external funding^1^  *Policy*  -Structural incorporation of intervention goals in policy^7^  -Principal facilitating policy implementation^18^  -National implementation of monitoring system^7^  -National certification of the program^7^ | **I. Community Level Factors**  *Prevention Theory and Research*  -Lack of evidence-based treatments for overweight/obese children^7^  *Funding*  -Shortage of financial resources^1^  *Policy*  -State requirements for mandated standards for other subjects^11^ |
| **II. Provider Characteristics**  *Perceived Need for Innovation*  -Matched the needs of the organisation^11^  -Perceived importance of participation/program goals^7^  *Perceived Benefits of Innovation*  -Perceived initial implementation as pilot; continued implementation builds on lessons learned^1^  -Continued implementation of perceived successful elements of the programme^1^ | **II. Provider Characteristics**  *Perceived Need for Innovation*  -Low priority relative to other academic subjects^11^  -Conflicting school commitments^14^  *Perceived Benefits of Innovation*  -Intervention perceived as project that stops after two years^1^  *Skill Proficiency*  -Need for improved match of tasks with regular functions and skills^7^ |
| **III. Characteristics of the Innovation**  *Compatibility*  -Alignment of Nutrition Services goals with intervention goal^14^  -Existing health-promoting programs were reinforced^14^  -Intervention materials ease of use^11^  *Adaptability*  -Flexibility of the intervention^18^  -Materials were collected, stored and re-used in schools^1^ | **III. Characteristics of the Innovation**  *Compatibility*  -Explicit wish for additional components aimed at healthy nutrition^7^ |
| **IV. Factors Relevant to the Prevention Delivery System: Organizational Capacity**  *Integration of new programming*  -High perceived fit into existing health promotion activities in the school^1^  *Shared vision*  -Shared program and school goals^14^  -Strong commitment of teachers/school management^1^  -Strong motivation to work with the programme^1^  *Coordination with other agencies*  -Continued participation of all partners^7^  -Formalisation of partnerships, networks, and collaborations^7^  *Formulation of tasks*  -Institutionalisation of intervention through new school traditions^14^  -Reallocation of tasks^7^  -Realistic expectations about tasks and responsibilities^7^  *Leadership*  -Program advisor working with parents, and student leadership^14^  *Managerial/supervisory/administrative support*  -Institutional support^14^  *Classroom management/disruptive student behaviour***^#^**  -Children being re-energised afterwards^17^ | **IV. Factors Relevant to the Prevention Delivery System: Organizational Capacity**  *Integration of new programming*  -Need for increased effective smart planning/controlled action^7^  -Lack of evaluation amongst teachers, no decision about continuation^1^  -Need for simplified methods, instruments, protocols, and tasks^7^  -Overload in curriculum/ schools prioritise other health promotion^1,7,17^  -Lack of time in the school day^11^  -Overlap with regular teaching materials^1^  *Coordination with other agencies*  -Complexity of multidisciplinary collaboration^7^  -Different financial structures of participating organisations^7^  -Complex financial structures because of multidisciplinary collaboration^7^  *Formulation of tasks*  -Lack of strategy for how to continue with intervention^1^  *Specific Staffing Considerations*  -Teacher turnover, student turnover, and re-placement of intervention in other subject, making a 2-year programme planning difficult^1^  -High teacher workload^1^  -Insufficient staffing^14^  -Staff reassignment^14^  *Leadership*  -Need for strong program management and centralised coordination^7^  -Change in school leadership^14^ |
| **V. Factors Related to the Prevention Support System**  *Training*  -Training (repeated over time, not just at the beginning)^11^ | **V. Factors Related to the Prevention Support System**  *Technical Assistance*  Lack of equipment and materials^11^  Lack of continuation due to loss of materials^1^ |
| **Others^#^**  *Student characteristics, engagement and motivation*  -Student interest/involvement^14^ | **Others^#^** |

*Studies represented by the following superscripts: ^1^(van Nassau et al. 2016a), ^2^(van Nassau et al. 2016b), ^3^(Mâsse et al. 2012), ^4^(McKay et al. 2015), ^5^(Nielsen et al. 2018a), ^6^(Nielsen et al. 2018b), ^7^(de Meij et al. 2013), ^8^(Saunders et al. 2011), ^9^(Bice, Brown & Parry 2014), ^10^(Franks et al. 2007), ^11^(Hoelscher et al. 2004), ^12^(Wiecha et al. 2004), ^13^(Graziose et al. 2017), ^14^(Beck, Jensen & Hill 2015), ^15^(Totura et al. 2015), ^16^(Chalkley et al. 2018), ^17^(Ryde et al. 2018), ^18^(Storey et al. 2011), ^19^(Austin et al. 2011), ^20^(Carlson et al. 2017).^#^Other categories as per the classification proposed by Naylor et al (14).
